# American Medical Association

**Published:** 1867-06

**Authors:** 


### AMERICAN MEDICAL ASSOCIATION.

It will be remembered that the American Medical Association was to meet at Cincinnati on the 7th ult., and that a few days previously, the Convention ot Colleges was to take place.

We give the following synopsis of the proceedings of the Association, from the Nashville Journal of Medicine and Surgery:

This body held its 18th Anniversary in Cincinnati, commencing on Tuesday the 11th, and continuing its sessions four days.

The reports of the daily proceedings were very meager and full of errors, as published by the press of that city—scarcely a name was spelt right. The entertainments given to the profession were on a magnificent scale, and lavished in a princely style. We have heard it said that they could not have cost less than one hundred thousand dollars.

The accounts received of the proceedings are not sufficiently accurate to make up a satisfactory report.

The Resolution of the Teachers' Convention held a few days previous to the meeting of the Association, were unanimously adopted.

Dr. Pickney read a lengthy report on Medical Rank in the Navy.

Dr. Post read the annual report on Medical Literature.

Dr. Walker, in the place of Dr. Ray, who was absent on account of illness, read the report of the Committee on Insanity.

A few other reports were made.

The Majority of the Standing Committees failed to report.

Professional and scientific subjects, when introduced, were referred to the various sections which the Association proposed to organize itself into each day. Of their doings we have as yet received no account, and apprehend that the papers, &c., offered to the Association, have been referred to the Committee on Publication, and subjected to their action alone, without discussion or even having been read.

The Resolutions of the Convention of Teachers of the Medical Colleges, approved and adopted by the Association, were:—

1st. Every student entering a Medical College must give evidence that he possesses a thorough knowledge of an English education, and understands Latin and Greek technical professional terms.

2d. Every medical student is required to study for four full years, including three annual courses of lectures.

3d. Six calendar months must be the duration of a regular lecture term.

4th. The Faculty shall be composed of not less than nine Professors, teaching Anatomy, Chemistry, Materia Medica, Toxicology, Pathology, Therapeutics, Physiology, Hygiene, Surgery, Medical Jurisprudence, Medical Ethics, Clinical Medicine and Surgery, Obstetrics, Diseases of women and children. These branches shall be divided into three groups or series, corresponding with the three courses of lectures.

5th. Every Medical College should adopt some effectual method of ascertaining that students are in attendance on the lectures.

There were twenty Colleges represented. The other institutions are to be called upon to take action on these resoulutions.

The following extracts from the St. Louis Medical and Surgical Journal, give not a very flattering account of these proceedings:

Dr. Davis, the father of the Association, arose; made a rather telling and vehement speech. He said that he expected nothing better for the Association; he expected the time to come when it would be invited *nowhere!* It had been badly conducted. There was too much eating and drinking and frolicking done. The expense of furnishing all this luxury was enough to frighten any city. He spoke of the great feast at St. Louis many years ago, in which some of the feasters became noisy and unruly. He said that very little had been done in the present meeting, either in full session or in sections. In short, the whole thing was rather a failure. So the Doctor introduced a resolution that the next meeting should be in the city of Washington, whither the Association had a right to go WITHOUT AN INVITATION. This is about all that need be said about the last meeting of the Association.

We understood that there was an excursion to Longworth's wine cellars after the adjournment. Let us see—yes, we have omitted one thing. Dr. Gross reported on Medical Education, and recommend three sessions instead of two, and six months instead of four or five per session, as necessary preliminaries to graduation. There was something else in the report about requiring mathematics. The whole was voted for without debate by some three or four hundred men, who had certainly devoted to it very little thought. The idea seemed to be that even the proposer of the GREAT REFORM regarded it impracticable, and that the action of the Convention *pro* or *con* was and would be a nullity, and that therefore it was just as well to "let it slide" *sub silentio.* We have no hesitation in saying, that we do not think the American Medical Association has done much for science or the elevation of the profession. The meetings look like "child's play," or a great annual frolic, which is rather unbecoming a grave and learned profession. It is pleasant, to be sure, to meet our professional brethren and friends, but the thing is too public; the festivities are too big and too often; the crown is too great, and we can not avoid the suspicion that the public is rather laughing at us "in its sleeve." Medical congresses, properly conducted, would be of vast service; but the number of delegates should be limited. The sessions should last for weeks, and men of ability should discuss the great questions of science to exhaustion, if possible. A volume of such discussions would be more sought after, we opine, than the "*Transactions*" of the American Medical Association.

